# Supplementary material for: DeepCOVID-Fuse: A Multi-Modality Deep Learning Model Fusing Chest X-rays and Clinical Variables to Predict COVID-19 Risk Levels
Source: Bioengineering (Basel). 2023 May 5;10(5):556. doi: 10.3390/bioengineering10050556 (PMC10215672; doi:10.3390/bioengineering10050556)
Supplement: Supplementary file 1 [file bioengineering-10-00556-s001.zip › bioengineering-2366850-supplementary.pdf]

bioengineering

MDPI

Supplemental Materials

DeepCOVID-Fuse: A Multi-Modality Deep Learning Model Fusing Chest X-Radiographs and Clinical Variables to Predict COVID-19 Risk Levels

Yunan Wu [1,*], Amil Dravid [2], Ramsey Michael Wehbe [3] and Aggelos K. Katsaggelos [1,2]

1 Department of Electrical and Computer Engineering, Northwestern University, Evanston, IL 60201, USA; aggk@eecs.northwestern.edu
2 Department of Computer Science, Northwestern University, Evanston, IL 60201, USA; amildravid2023@u.northwestern.edu
3 The Division of Cardiology, Department of Medicine and Bluhm Cardiovascular Institute, Northwestern Memorial Hospital, Chicago, IL 60611, USA; ramsey.wehbe@northwestern.edu
* Correspondence: yunanwu2020@u.northwestern.edu

1. DeepCOVID-Fuse

The architecture of the ensemble fusion model is shown in Figure 1. The ensemble was chosen because studies have shown that the ensemble architecture is able to improve model performance and generalization ability [4]. Different combinations of feature dimensions from the image branch and the clinical variable branch when concatenated were compared. The feature dimension of the clinical variable branch after a fully connected layer was fixed at 128 while the feature dimension of the image branch varied, including 64 (smaller than clinical features), 128 (equal to clinical features), and the same dimension after each model's average pooling layer (larger than clinical features).

2. Image-Only Model and Feature-Only Model

The image-only model was trained and tested only on CXRs images, which used the image branch of the fusion model. As shown in Figure 1, after the dropout layer of the image branch, the features were fed into the dense layer without the concatenation layer. Similarly, the feature-only model trained and tested only on clinical variables, which used the clinical branch of the fusion model, i.e., after the dropout layer of the clinical branch, the features were fed into the dense layer without the concatenation layer.

3. Fusion-Image-Only and Fusion-Feature-Only Model

The Fusion-image-only model used the well-trained DeepCOVID-Fuse model (i.e., the trained weights), but only used CXRs as input for testing. Although no clinical variables were used, the fusion model required them as input, so we computed the average clinical features from the training set and used them as input to the test set, which means all test sets had the same clinical features calculated from the training set.

The Fusion-feature-only model used the same well-trained DeepCOVID-Fuse model but only used clinical features as input for testing. The fusion model still required a CXR input, so we chose a CXR image from the training set that had the most equal probability for the three classes predicted by the model. In other words, we did not want the images to add any bias to the fusion model predicted from clinical features alone.

3. Model Implement Details

Each individual architecture first initialized the weights from [4] (GitHub: [the link for the weights]). The convolutional layers were frozen, and the model was trained to fine-tune all fully connected layers and the clinical variable branch. Then, all layers were unfrozen and trained again on the training set. We used stochastic gradient descent as the

optimizer with initial learning of 0.0002 and momentum of 0.9. The batch size was set as 16 and early stopping with patience of 8 was used to avoid overfitting. The loss function was the class-weighted categorical cross-entropy loss, which was defined as:

$$L = \sum_i -\alpha_i y_i log\ (p_i) \quad \text{(S1)}$$

Where y_i is the ground truth class of each subject i, p_i is the predicted probabilities from the model, and α_i is the class weight, which is calculated by inverting the frequency of each class to alleviate the class imbalance problem. Our code is publicly available on GitHub at https://github.com/YunanWu2168/DeepCOVID-Fuse.

Table S1. Patient characteristics from the training, validation, and test set.

Clinical Variables	Training	Validation	Test
Total (n)	1657	428	439
Gender (M/F)	850/807	238/190	234/205
Age (mean ± std)	58.30 ± 17.74	56.41 ± 17.03	56.51 ± 17.78
RACE (n)			
Asia	78	7	10
Black or African American	436	112	60
White	856	234	325
Other	234	64	32
Unknown	53	11	12
ETHNIC (n)			
Hispanic or Latino	381	127	256
Not Hispanic or Latino	1231	290	176
Other	45	11	7
SMOKING STATUS (n)			
Never smoker	877	245	300
Smoker	586	137	120
Unknown	199	46	19
Atrial Rate	91.27 ± 31.95	95.45 ± 37.95	98.01 ± 35.62
ECG (mean ± std)			
P-R Interval	164.05 ± 26.45	155.22 ± 27.92	154.32 ± 27.90
QRS Duration	91.58 ± 20.68	90.31 ± 19.11	88.48 ± 17.16
QT	376.35 ± 49.57	369.93 ± 50.00	359.22 ± 41.55

QTC	448.81 ± 35.93	444.39 ± 36.73	444.93 ± 30.49
BP Diastolic	76.89 ± 14.85	75.75 ± 14.02	78.15 ± 14.12
BP Systolic	136.32 ± 22.60	134.83 ± 22.63	135.87 ± 22.47
Pulse	93.76 ± 18.93	96.30 ± 20.63	99.01 ± 18.45
Respirations	20.99 ± 5.16	21.58 ± 5.24	23.40 ± 7.33
SPO2	95.11 ± 6.69	94.83 ± 5.09	92.45 ± 10.41
Temperature	99.15 ± 1.50	99.32 ± 1.48	99.96 ± 1.61
Comorbidity (n)			
Asthma	4	2	0
COPD	249	38	24
Cancer	72	8	21
Cardiovascular	654	156	113
Cerebrovascular disease	285	65	49
Chronic pulmonary disease	543	131	96
Congestive heart failure	378	78	41
Dementia	177	43	55
Diabetes	614	170	191
HIV_AIDS	39	27	4
Hemiplegia or paraplegia	58	18	10
Hypertension	1026	251	240
Immunological	105	37	18
Malignancy	239	51	62
Metastatic Solid Tumor	126	25	26
Myocardial infarction	131	23	17
Peptic ulcer disease	90	15	16
Peripheral vascular disease	228	44	40
Renal disease	538	120	137
Rheumatic disease	74	31	13
liver disease	206	36	78

LAB (mean ± std)			
A Eos percent	0.84 ± 1.66	0.63 ± 1.33	0.68 ± 1.52
Absolute basophils	0.01 ± 0.03	0.01 ± 0.03	0.01 ± 0.03
Absolute eosinophils	0.05 ± 0.12	0.05 ± 0.12	0.07 ± 0.43
Absolute immature granulocytes, automated	0.05 ± 0.10	0.04 ± 0.07	0.05 ± 0.09
Absolute lymphocytes	1.38 ± 2.53	1.35 ± 0.90	1.22 ± 0.79
Absolute monocytes	0.58 ± 0.83	0.52 ± 0.26	0.56 ± 0.33
Absolute neutrophils	5.41 ± 4.79	5.36 ± 3.34	5.98 ± 3.79
Albumin	3.82 ± 0.52	3.77 ± 0.47	3.72 ± 0.42
Alkaline phos	79.84 ± 49.65	74.65 ± 41.12	85.26 ± 55.43
ALT (SGPT)	39.80 59.28	43.36 ± 50.74	38.44 ± 35.16
Anion gap	12.06 ± 3.47	11.86 ± 3.48	10.72 ± 3.19
AST (SGOT)	48.17 ± 89.00	48.68 ± 47.42	43.26 ± 38.67
Basophils	0.19 ± 0.41	0.18 ± 0.40	0.14 ± 0.35
C-Reactive protein	38.77 ± 60.38	29.75 ± 47.82	31.93 ± 51.42
Calcium	8.91 ± 0.60	8.93 ± 0.75	8.90 ± 0.59
Chloride	100.68 ± 5.04	101.07 ± 5.18	99.43 ± 5.38
CO_2	23.80 ± 3.68	23.72 ± 3.65	24.83 ± 3.56
Creatinine	1.38 ± 1.68	1.43 ± 1.42	1.23 ± 1.37
D-dimer	1105.15 ± 3456.19	1199.95 ± 5898.18	1635.64 ± 6804.57
Direct bilirubin	0.21 ± 0.20	0.20 ± 0.28	0.20 ± 0.21
Eosinophils	0.82 ± 1.57	0.77 ± 1.33	0.94 ± 1.66
GFR (African American)	70.13 ± 35.81	66.05 ± 30.42	55.31 ± 12.02
GFR (others)	62.80 ± 28.63	58.85 ± 24.83	53.65 ± 13.33
Glucose	143.93 ± 78.06	141.58 ± 70.19	155.08 ± 82.29
Hematocrit	39.71 ± 5.58	39.53 ± 5.52	39.69 ± 5.57
Hemoglobin	13.05 ± 2.04	13.00 ± 2.07	13.30 ± 2.08
Immature granulocytes	0.62 ± 0.99	0.50 ± 0.68	0.63 ± 0.64
INR	1.26 ± 0.67	1.66 ± 1.84	1.25 ± 0.44

LDH	383.11 ± 418.68	343.41 ± 146.85	332.39 ± 168.69
Lymphocytes	19.79 ± 11.10	20.34 ± 11.81	17.78 ± 10.07
MCH	28.98 ± 2.58	28.85 ± 2.37	29.39 ± 2.45
MCHC	32.80 ± 1.46	32.82 ± 1.54	33.44 ± 1.41
MCV	88.31 ± 6.41	87.93 ± 5.86	87.91 ± 6.49
Monocytes	8.32 ± 4.21	7.70 ± 3.75	7.70 ± 3.81
MPV	10.38 ± 0.96	10.34 ± 0.90	10.32 ± 0.94
Neutrophils	70.03 ± 13.62	70.46 ± 13.30	72.91 ± 12.32
Platelet count	224.41 ± 87.40	233.81 ± 94.25	227.47 ± 95.41
Potassium	4.03 ± 0.61	3.99 ± 0.56	3.81 ± 0.50
Procalcitonin	0.73 ± 4.05	0.83 ± 5.65	0.81 ± 4.48
Prothrombin time (PT)	14.53 ± 8.17	19.39 ± 21.93	14.09 ± 5.17
RDW	13.88 ± 1.99	13.84 ± 1.97	13.65 ± 1.80
Red cell count	4.53 ± 0.74	4.52 ± 0.71	4.54 ± 0.72
Sodium	136.54 ± 4.20	136.66 ± 4.18	134.68 ± 4.53
Total bilirubin	0.61 ± 0.44	0.55 ± 0.29	0.65 ± 0.63
Total protein	7.12 ± 0.76	7.06 ± 0.71	7.29 ± 0.66
Troponin-I	0.15 ± 1.04	0.03 ± 0.05	0.04 ± 0.12
Urea Nitrogen	19.44 ± 15.77	20.99 ± 15.93	19.98 ± 20.35
White blood cell count	7.73 ± 11.04	7.33 ± 3.58	7.96 ± 5.15

Table S2. Performance of DeepCOVID-Fuse (Ensemble) for risk predictions in confirmed COVID-19 subjects on external test sets in different age groups.

**Ensemble Results in Different Age Groups**	**20–40**	**40–60**	**60–80**	**80–100**	**All**
Size	80	186	119	54	439
Distribution	L:32, IM:29, H:19	L:52, IM:87, H:47	L:14, IM:54, H:51	L:3, IM:23, H:28	L:101, IM:193, H:145
Accuracy	0.652	0.661	0.692	0.632	0.658
Recall	0.650	0.662	0.689	0.627	0.660
Precision	0.688	0.677	0.699	0.618	0.689
F1	0.642	0.660	0.863	0.620	0.660

MCC	0.623	0.638	0.645	0.604	0.640
AUC	0.821	0.845	0.847	0.791	0.842

Note.—AUC = area under the receiver operating characteristic curve; L = low risk level; IM = intermediate risk level; H = high risk level.

Table S3. Performance of fusion-image-only models for risk prediction in confirmed COVID-19 subjects on external test sets using CXRs as model input with a random subset (%) of clinical variables (0: no clinical features, 100: full clinical features).

**COVID-Level (Proportion of Clinical Features in Fusion)**	**0**	**20%**	**40%**	**60%**	**80%**	**100%**
Accuracy	0.623 [0.604, 0.641]	0.625 [0.615, 0.635]	0.630 [0.620, 0.640]	0.635 [0.620, 0.650]	0.645 [0.634, 0.656]	0.658 [0.650–0.667]
Recall	0.620 [0.608, 0.632]	0.623 [0.607, 0.638]]	0.628 [0.617, 0.639]]	0.634 [0.615, 0.653]	0.647 [0.635, 0.657]	0.657 [0.649–0.666]
Precision	0.639 [0.623, 0.647]	0.637 [0.619, 0.655]]	0.640 [0.622, 0.658]	0.647 [0.632, 0.662]	0.656 [0.643, 0.669]	0.671 [0.658, 0.684]
F1	0.627 [0.611, 0.639]	0.627 [0.612, 0.642]	0.630 [0.614, 0.645]	0.634 [0.619, 0.649]	0.644 [0.633, 0.656]	0.658 [0.650, 0.666]
MCC	0.613 [0.605, 0.621]	0.612 [0.601, 0.623]	0.616 [0.607, 0.625]	0.623 [0.614, 0.631]	0.625 [0.618, 0.632]	0.635 [0.629, 0.641]
AUC	0.797 [0.784, 0.806]	0.798 [0.787, 0.809]	0.804 [0.796, 0.812]	0.808 [0.802, 0.814]	0.816 [0.814, 0.818]	0.824 [0.822, 0.826]

Note.—Data in parentheses are 95% CIs from five repeated experimental runs. AUC = area under the receiver operating characteristic curve; fusion-image-only = well-trained fusion models but tested with CXRs only.

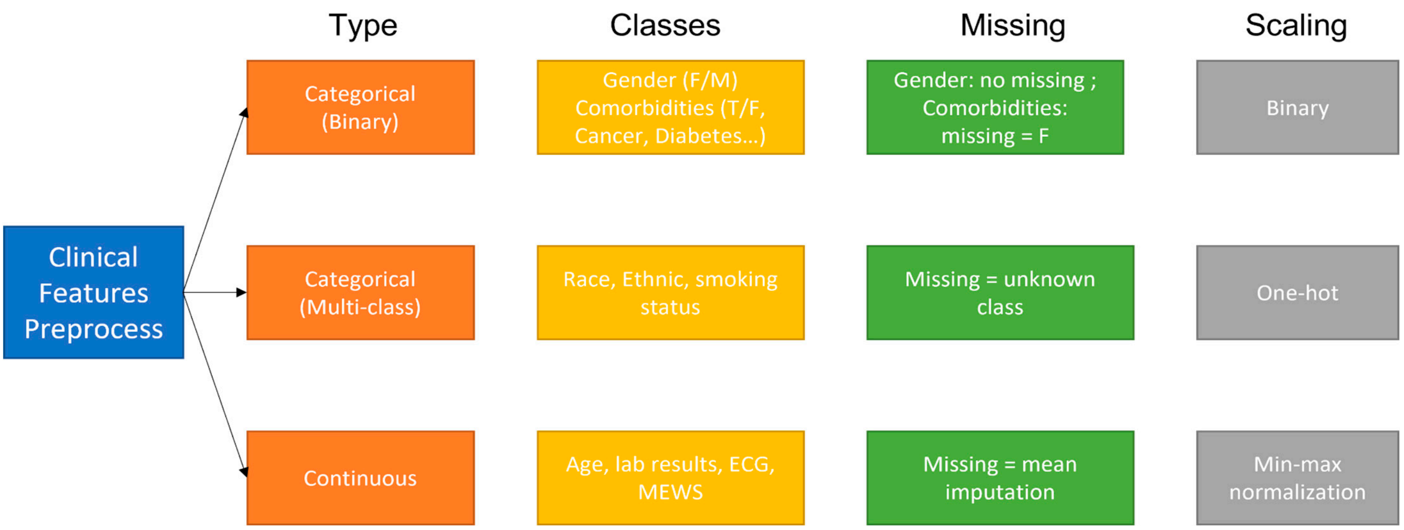

Figure S1. The preprocessing of clinical features. Features are classified into three types, binary, multi-class, and continuous with different missing imputation and scaling operations.
